# Evaluation of geographic variations in appendicectomy outcomes within Western Australia assessing the impact of surgical wait times and rate of negative appendicectomies in both urban and rural locations statewide

**DOI:** 10.1007/s00384-024-04662-3

**Published:** 2024-06-13

**Authors:** Beau Scaddan, Balsam Al Asedy, Samantha Lee, Parsa Rastegar Lari

**Affiliations:** 1 Curtin Medical School, Perth Western Australia, Perth, Australia; 2 UWA Medical School, Perth Western Australia, Perth, Australia

**Keywords:** Appendicectomy, Appendectomy, General surgery, Acute abdomen, Appendicitis

## Abstract

**Purpose:**

Surgery wait times after diagnosis of appendicitis are an important factor influencing the success of a patient’s treatment. The proposed study will be a quantitative multicenter retrospective cohort design with the primary aim of assessing the difference between appendicectomy wait times between rural and urban hospitals in Western Australia and the effect of this on operative outcomes. Selected outcome measures will be examined by time from initial presentation at an emergency department to the patient being diagnosed and then time of diagnosis to surgery being performed. The secondary aim is to compare rates of negative appendicectomies between hospitals.

**Methods:**

Appendicectomy patients will be identified from operating room register by medical student data collectors; then, each respective hospital’s emergency room data collection will subsequently be accessed to complete case report forms based on demographics and clinical findings, pre-operative investigations, and management and follow-up. Case report forms with > 95% completeness will be accepted for pooled analysis. The expected duration of retrospective data collection will be 8 months. This study RGS6483 has received HREC approval by the Royal Perth Hospital HREC Ethics Committee, with a waiver of consent obtained and the HREC was notified of amendments to the protocol made on April 21, 2024.

Dissemination of results.

Data will be collected and stored online through a secure server running the Research Electronic Data Capture (REDCap) web application. No patient-identifiable data will be entered into the system. Results will subsequently be shared via scientific journal publication and presentation at relevant meetings.

**Supplementary information:**

The online version contains supplementary material available at 10.1007/s00384-024-04662-3.

## Introduction

Surgery wait times after diagnosis of appendicitis are an important factor influencing the success of a patient’s treatment. Clinical evaluation in conjunction with the Alvarado or appendicitis inflammatory response scores is used to determine the risk of appendicitis in symptomatic patients. A large cohort study has shown that, in cases of uncomplicated appendicitis, short delays to operation of < 24 h do not increase the rates of complex appendicitis [1]. This finding was confirmed in a meta-analysis of 11 non-randomized studies, where a short in-hospital delay of 12 to 24 h before surgery did not increase the risk of complex appendicitis including perforation. However, delaying appendicectomy for > 48 h was associated with increased surgical site infections and other complications [2]. A laparoscopic appendicectomy approach should be performed unless contraindicated [3].

Negative appendicectomy refers to surgical removal of the normal appendix on final pathological report [4]. The Second Australian Atlas of Healthcare Variation in 2017 revealed that negative appendicectomy rates in Australia were 21–24% [5]. With appendicectomies carrying many risks such as possible wound infection or rupture, intestinal injury, postoperative abdominal abscess, or urinary infections, unnecessary appendicectomy can have significant impacts [6]. A recent audit conducted to examine pediatric negative appendicectomy rates at Southwest Health Campus Bunbury including 421 patients found the negative appendicectomy rate to be higher than observed in previous literature at 45.58%. The authors noted this was concerning since diagnostic laparoscopy in children is not benign, with negative appendicectomy having a similar morbidity risk to appendicectomy for uncomplicated appendicitis [7].

The proposed study will be a quantitative multicenter retrospective cohort design with the primary aim of assessing the difference between appendicectomy wait times between rural and urban hospitals in Western Australia and the effect of this on selected outcome measures. The secondary aim is to compare the rates of negative appendicectomies between these hospitals.

It is hypothesized that there will be a higher rate of negative appendicectomies in rural hospitals as opposed to metropolitan hospitals, rooting from various potential factors. According to an audit examining the rural–urban differences in surgical interventions in the United States (US), there were higher rates of an array of surgeries including appendicectomies in areas classified rural according to US standards. This was contrary to the authors’ hypothesis that rurally located patients would have lower surgical rates. The authors proposed this could be explained by rural patients experiencing overall poorer health, increasing the indications for many surgeries to be undertaken [8]. This can be extrapolated to the Australian setting, where, according to the Australian Institute of Health and Welfare (AIHW), risk factors such as smoking, obesity, and hypertension amongst many others all increased with rurality, as well as chronic health conditions and burden of disease [9]. Another factor which may contribute to potential higher negative appendicectomies is that doctors may have a lower threshold to perform the surgery in order to avoid potential severe complications, such as perforation in a setting where there is reduced healthcare access. This may in turn lead to reduced surgical wait times in rural settings, addressing both our primary and secondary outcomes. Another US study on pediatric patients revealed that rural hospitals were 50% more likely to perform negative appendicectomies. The authors explained this is due to negative appendicectomies being justified in order to “decrease delay in diagnosis and subsequently lower the risk of appendiceal perforation” [10]. The authors noted that this disparity may also be in part due to reduced access to pre-operative imaging such as computed tomography to make the diagnosis. It is suspected a combination of all stated factors may play a role in reduced surgical wait times and negative appendicectomies in rural compared to metropolitan hospitals in Western Australia.

## Aim

The aim of this study was to determine the variation in appendicectomy surgery wait times between rural and urban areas in Western Australia after initial presentation and diagnosis at an emergency department. Within these populations, selected outcome measures will be examined by time from:

1. Initial presentation for the presenting complaint at an emergency department to the patient being seen by a general surgery registrar or consultant.
2. Time from patient being seen by a general surgery registrar or consultant to surgery being performed.

The reason for taking data based on the two separate time frames is that by recording time between initial emergency department presentation to the patient being seen by the general surgery registrar or consultant, it will be possible to account for those patients who are not immediately diagnosed and then go on to have a delayed diagnostic laparoscopy and appendicectomy and evaluate and measure the magnitude of outcome impacts that an increased duration of this time could produce. Furthermore, the case report forms collect data on previous presentations of the same presenting complaint within the past week to capture any *prior premature discharge from the same site on initial evaluation of presenting complaint*.

This will allow examination of the effect of both triage wait times and timing of surgery after diagnosis on perforation, operation duration, and length of stay amongst each of the subpopulations.

Additionally, it is hoped to establish the rate of negative appendicectomies in urban and rural areas in Western Australia and if necessary make suggestions preoperatively to reduce their incidence.

## Audit standards for outcome measures

The optimal timing of appendicectomy for uncomplicated acute appendicitis should be within 12 h of initial presentation at an emergency department [1].

Laparoscopic appendicectomy approach should be performed unless contraindicated [3].

### Primary outcome measure

Wait time from presentation (hours) to diagnosis and then surgery. This is the number of hours from initial presentation at an emergency department until review by the surgical team, diagnosis, and then surgery. These parameters will be recorded on the case report forms as date and time and will be used to compare such wait times between rural and metropolitan hospitals.

### Secondary outcome measures

#### Rate of laparoscopic appendicectomy

This is the number of appendicectomies performed in rural and metropolitan hospitals. This will allow us to explore the overall rate compared to rates of negative appendicectomies in the specified hospitals.

#### Rate *of complex* appendicitis

This is the rate of appendicitis deemed complex based on various parameters. There are many grading systems such as the Sunshine Appendicitis Grading System (SAGS) as well as classification into simple (non-perforated) and complex (gangrenous or perforated) by Bhangu et al. [11]. Due to the retrospective nature of this study, it is impossible to determine whether all cases underwent intra-operative classification of severity using the such scoring. To address this, the case report forms in this study have been adapted to contain a macroscopic (physical) and microscopic (histological) evaluation of the case, which is more likely to have taken place and provides the same information.

##### Hospital stay length

This is the number of days that patients will be remaining in hospital from the date of their initial presentation in the emergency department. This will provide us with further information on the complexity of the cases as well as the post operative health needs of the patients undergoing appendicectomies.

##### Operation duration

This is the duration in hours of the appendicectomies performed in rural and metropolitan hospitals. This again will provide general information about the complexity of cases performed.

##### Rate of negative appendicectomy

This is the rate of appendicectomies performed in normal appendices. This is reported on the case report forms under macroscopic and microscopic appearance, as a normal appendix or simple or complex appendicitis. This will allow us to compare the rates of negative appendicectomies between rural and metropolitan hospitals and provide us with data to examine the potential reasons behind this, outlined in the “Introduction” section.

## Methods

This protocol adheres to the reporting recommendations relevant specifically to an observational retrospective cohort study of the Guidelines for Reporting Outcomes in Trial Protocols: The SPIRIT-Outcomes (2022), obtained via the Equator Guidelines [12]. Consultation with doctors in the field of general surgery and rural health experts from WACHS was performed in the development of this protocol to ensure that the needs of rural populations are not misrepresented.

The study design was chosen to be retrospective due to the difficulties of prospective recruitment by medical student data collectors for whom it is unrealistic to conduct a prospective study to recruit appendicitis patients due to the transient nature of placements, especially in large metro hospitals, which would likely lead to selection bias (for more details, see “Collaborative teams”). Four separate 2-week data collection periods were preferred over one asynchronous period of data collection to provide structure for the data collection into four 6-month retrospective review periods which would allow more medical students to participate in data collection in the project overall if each student is allocated one data collection period and to divide the amount of retrospective data to be reviewed into a manageable amount for each mini-team (for more details see “Collaborative teams”).

The retrospective time period which will be examined for eligible patients and from which data is collected will be over a 2-year period from January 2022 to November 2023. Appendicectomy patients will be identified from the operating room register by members of the mini-teams discussed in the “Methods” section, expected to be mainly medical student data collectors currently on placement at the relevant hospital, and then, each respective hospital’s emergency room data collection subsequently will be accessed to fill out the case report forms. The data will be collated via case report forms based on demographics and clinical findings, pre-operative investigations, and management and follow-up. Case report forms with > 95% completeness will be accepted for pooled analysis. The expected duration of retrospective data collection will be 8 months. This includes the four 2-week data collection periods.

The planned first data collection period will only record information for eligible patients receiving appendicectomy at the relevant center between January and June 2022 retrospectively to their surgeries being performed, and then, a check of the patient’s record until the 30-day postoperative interval on the same data collection source will be conducted to record any readmissions or complications.

The planned second data collection period will only record information for eligible patients receiving appendicectomy at the relevant center between June 2022 and December 2022 retrospectively to their surgeries being performed, and then, a check of the patient’s record until the 30-day postoperative interval on the same data collection source will be conducted to record any readmissions or complications.

The planned third data collection period will only record information for eligible patients receiving appendicectomy at the relevant center between January 2023 and June 2023 retrospectively to their surgeries being performed, and then, a check of the patient’s record until the 30-day postoperative interval on the same data collection source will be conducted to record any readmissions or complications.

The planned fourth data collection period will only record information for eligible patients receiving appendicectomy at the relevant center between June 2023 and November 2023 retrospectively to their surgeries being performed, and then, a check of the patient’s record until the 30-day postoperative interval on the same data collection source will be conducted to record any readmissions or complications.

For the purpose of this project, sites under WA Country Health Service (WACHS) jurisdiction will be considered rural, that is, Albany Hospital, Bunbury Hospital, Broome Health Campus, Geraldton Hospital, and Hedland Health Campus. The other sites (Royal Perth Hospital, Sir Charles Gairdner Hospital, Fiona Stanley Hospital, Armadale Health Service) will be considered urban.

Following data collection, only case report forms with > 95% data completeness will be accepted for pooled analysis. Centers with > 5% missing data points will be excluded and collaborators from those centers withdrawn from the published list of citable collaborators. This is in order to provide some leeway for potential missing data, while also acknowledging that case report forms with ≥ 5% missing data may not be useful for our purposes. As the data originates from emergency department records, there is no perceived risk of including false or unused data. This study RGS6483 has received HREC approval by the Royal Perth Hospital HREC Ethics Committee, with a waiver of consent obtained and the HREC was notified of amendments to the protocol made on April 21, 2024.

### Collaborative teams

#### Steering committee

A group of medical students whose role is to design the protocol, final dataset handling and analysis and drafting of the paper.

#### Mini-teams

Mini-teams consist of five collaborators at each site whose role is to identify eligible patients receiving appendicectomies during the study periods and collect data. The mini-teams are planned to consist mainly of student data collectors who are currently on placement at the relevant hospital, recruited through the student research network STRIVE WA. One collaborator is to be selected as the local lead. For every center, there is to be a local lead in the form of a surgical consultant, who coordinates and supervises the actions of that center. All collaborators part of a mini-team whose data is accepted for the final pooled analysis will be listed as co-authors on the final manuscript.

### Sample size

There are over 1600 participants that are planned to be included. This estimate was obtained from the Australian Government Medical Statistics Record, which displayed 818 WA claims for MBS item number 30720, the code for the surgical operation to be examined within this study from July 2022 to June 2023. Assuming this is representative for the beginning of 2022 to the end of 2023, the study window for this project, the number of participants can reasonably be estimated to be double this figure.

Amongst the metropolitan hospitals (Royal Perth Hospital, Sir Charles Gairdner Hospital, Fiona Stanley Hospital and Armadale Health Service), there will be 350 participants each, and at the rural hospitals (Albany Hospital, Bunbury Hospital, Broome Health Campus, Geraldton Hospital and Hedland Health Campus), there will be 50 participants each. This is reflective of the respective sizes of the hospitals.

### Data collection and storage

Data will be collected and stored online through a secure server running the Research Electronic Data Capture (REDCap) web application. REDCap allows collaborators to enter and store data in a secure system. Collaborators will be given individual REDCap login details, allowing secure data storage on the REDCap system. No identifiable patient information will be collected. Collaborators may wish to first record data on a paper version of the data collection pro-forma. Paper copies of any data should be destroyed as confidential waste within the center once uploaded to REDCap.

Identifiable patient information will not be entered into REDCap since only the case report forms will be completed for every eligible instance of appendicectomy surgery.

Essentially, this study involves nothing from the participants themselves at all, only a retrospective review of their surgery admission. A report of the study will be sent to a medical journal approximately 4 months following completion of data analysis, and due to patient anonymity, they will not be informed of the outcomes of the project.

### Criteria

Inclusion criteria:

(i) All patients who undergo an appendicectomy during the study period.
(ii) Age 16 years or older at time of presentation

Exclusion criteria:

(i) Previous appendicectomy or right hemi-colectomy or total colectomy.
(ii) Current pregnancy at time of presentation

### Data analysis

Normally distributed data will be reported as mean (standard deviation (SD)), and non-normally distributed data will be reported as median (interquartile range (IQR)). Independent *t*-tests or ANOVAs will be used for normally distributed variables, Mann–Whitney *U* and Kruskal–Wallis tests will be used for non-normally distributed continuous or ordinal variables, and chi-squared tests will be used for categorical variable comparisons. When comparing appendicectomy wait times with complication incidence during the data analysis phase, a *P* value of 0.05 will be used to determine statistical significance. There will be no interim analysis or audit planned due to the grouping of data sets over four data collection periods. As this is a retrospective observational cohort study, there are no plans to deviate from the original protocol statistical plan or study design.

## Supplementary Information

Below is the link to the electronic supplementary material.

Supplementary file1 (DOCX 40.1 KB)

Supplementary file2 (PDF 359 KB)

## Data Availability

The full Redcap database will not be made publicly available however detailed data analysis will be published in the final report. Results will subsequently be shared via scientific journal publication and presentation at relevant meetings.
