# Supplementary material for: Evaluation of geographic variations in appendicectomy outcomes within Western Australia assessing the impact of surgical wait times and rate of negative appendicectomies in both urban and rural locations statewide
Source: Int J Colorectal Dis. 2024 Jun 13;39(1):91. doi: 10.1007/s00384-024-04662-3 (PMC11169051; doi:10.1007/s00384-024-04662-3)
Supplement: Supplementary file 1 — Supplementary file1 (DOCX 40.1 KB) [file 384_2024_4662_MOESM1_ESM.docx]

**CASE REPORT FORMS:**

**Case Report Form A – Demographics and Clinical Findings**

| **Biological sex** | ☐Male | ☐ Female |  |  |
| --- | --- | --- | --- | --- |
| **Age** | _____ years |  |  | Age in years on day of admission |
| **History of previous abdominal surgery** | ☐ Yes | ☐ No |  | Include open and laparoscopic surgery |
| **Previous acute inpatient admission with RIF pain within past week** | ☐ No previous admissions | ☐ Yes – one previous admission | ☐ Yes – two or more previous admissions | Any previous hospital presentation with RIF pain |
| **Date and time that patient was referred for initial review** | ___/___/___ ___:___ | | | Initial review is the first time the patient is seen by a member of the surgical team |
| **Date and time of initial review** | ___/___/___ ___:___ | | | As above |
| **Duration of symptoms** | ☐ <24 hours | ☐ 1 day | ☐ 2 days | Time from onset of symptoms to presentation at hospital on this admission |
|  | ☐ 3 days | ☐ 4 days | ☐ 5 days |  |
|  | ☐ 6 days | ☐ 7+ days |  |  |
| **How was this patient referred to the surgical team** | ☐ Emergency dept referral | | ☐ Inpatient referral |  |
|  | ☐ Transfer from another hospital, name: _______________________________________ | | |  |
|  | ☐ GP referral | | |  |
| **Was an appendicitis risk score used by the clinical team** | ☐ No | ☐ Yes – Alvarado | ☐ Yes – AIR | AIR = Appendicitis Inflammatory Response score |
|  | ☐ Yes – Other: | | |  |
| **If applicable, what was the risk score** | ______ | | |  |
| **Clinical findings at presentation (answer ‘no’ unless specifically noted)** | | | | |
| **Nausea** | ☐ Yes | ☐ No |  |  |
| **Vomiting** | ☐ Yes | ☐ No |  |  |
| **Anorexia** | ☐ Yes | ☐ No |  | Loss of appetite |
| **RIF pain** | ☐ Yes | ☐ No |  |  |
| **Migration of pain to RIF** | ☐ Yes | ☐ No |  | Migration from central abdomen to RIF |
| **RIF examination findings** | ☐ No tenderness | ☐ Tender but no guarding | ☐ Localised guarding |  |
|  | ☐ Generalised guarding |  |  |  |
| **RIF rebound tenderness** | ☐ Yes | ☐ No | ☐ Not tested |  |
| **Rosving’s sign** | ☐ Positive | ☐ Negative |  | Positive = palpation in left lower quadrant increases the pain felt in right lower quadrant |
| **Temperature on admission** | _____ °C | | |  |

**Case Report Form B – Pre-operative Investigations**

| **Date and time seen by General Surgeon or Consultant** | ___/___/___ ___:___ |  |  |  |
| --- | --- | --- | --- | --- |
| **Pre-operative Imaging** |  |  |  | Appendicitis confirmed: scan reported as demonstrating inflamed appendix.    Appendicitis ruled out: scan reported as demonstrating normal appendix.    Equivocal: scan reported as being unable to either confirm or disprove a diagnosis of appendicitis with any certainty. |
| **Ultrasound (US)** | ☐ Not performed | Findings (see codes below):_______ |  |  |
| **Appendicitis on US** | ☐ Appendicitis confirmed | ☐ Equivocal | ☐ Appendicitis ruled out |  |
|  | ☐ Not applicable |  |  |  |
| **Computed Tomography (CT)** | ☐ Not performed | Findings (see codes below):_______ |  |  |
| **Appendicitis on CT** | ☐ Appendicitis confirmed | ☐ Equivocal | ☐ Appendicitis ruled out |  |
|  | ☐ Not applicable |  |  |  |
| **Diagnostic Codes: For Imaging (CRF B) and Discharge Diagnosis (CRF C)** | (1) Appendicitis | (2) Colorectal cancer | (3) Crohn’s Disease |  |
|  | (4) Gastroenteritis | (5) Hernia | (6) Intrabdominal abscess |  |
|  | (7) Meckel’s Diverticulum | (8) Mesenteric Adenitis | (9) Mesenteric Thrombosis |  |
|  | (10) Urinary tract infection | (11) Renal calculi | (12) Ectopic pregnancy |  |
|  | (13) Endometriosis | (14) Ovarian cyst | (15) Pelvic inflammatory disease |  |
|  | (16) Non-specific pain | (17) Other: please specify |  |  |

**Case Report C – Management and Follow Up**

| **Was the patient transferred from your centre to another centre for further management, having not had any surgery at your centre?**  ***If so stop data collection at this point*** | ☐ No | | | ☐ Yes – admitting hospital does not offer acute surgery for this age group |  |  |
| --- | --- | --- | --- | --- | --- | --- |
|  | ☐ Yes – transferred to tertiary centre due to patient comorbidity (eg dialysis/transplant patient) | | | | |  |
|  | ☐ Yes – transferred to tertiary centre due to severity of presenting illness | | | | |  |
|  | ☐ If yes, hospital name: ____________________________________________ | | | | |  |
| **Was an operation performed** | ☐ No | ☐ Yes – index admission | | | ☐ Yes – on readmission | Index admission is the first presentation to hospital |
| **Were antibiotics administered as sole treatment of appendicitis?** | ☐ Yes | ☐ No | | |  |  |
| **Date and time of operation** | ___/___/___ ___:___ | ☐ Not applicable | | |  |  |
| **Main discharge diagnosis** | __________ (main diagnosis for index admission - please use diagnostic codes from previous page) | | | | |  |
| **Length of stay of index admission** | _______ days | | | | |  |
| **Re-admission to hospital**  ***With 30 days of index admission date*** | ☐ No | | | | | If applicable, select both ‘yes’ options |
|  | ☐ Yes – not operated on previous admission; re-admitted with ongoing RIF pain | | | | |  |
|  | ☐ Yes – operated on previous admission; re-admitted with post-operative complication | | | | |  |
| **Operative details and histology** | | | | | | |
| **What was the highest grade of surgeon who saw patient prior to surgery** | ☐ Consultant | ☐ Service Registrar | | | ☐ Surgical trainee |  |
|  | ☐ RMO/Intern |  | | |  |  |
| **How long did the operation take** | _____ hours_____ minutes | | | | |  |
| **Operative approach** | ☐ Laparoscopic | ☐ Lap converted to open | | | ☐ Open RIF incision |  |
|  | ☐ Open midline incision |  | | |  |  |
| **If initial open approach, reason** | ☐ Patient Preference | ☐ Trainee Preference | | | ☐ Consultant Preference | Include equipment availability under ‘theatre capability’ |
|  | ☐ Local policy for adults | ☐ Local policy for children | | | ☐ Theatre capability |  |
|  | ☐ Disease severity | ☐ Patient comorbidity | | | ☐ Previous surgery |  |
| **Procedure(s) completed** | ☐ Diagnostic only | ☐ Appendicectomy | | ☐ Caecectomy | ☐ Right hemicolectomy | Further details of the operation performed may be recorded in free text, if required |
|  | ☐ Meckle’s resection | ☐ Small bowel resection | | | ☐ Ileocolic resection |  |
|  | ☐ Other surgery (please specific):___________ | | | | |  |
| **Re-intervention following surgery**  **With 30 days of index admission date** | ☐ No | ☐ Yes – radiological re-intervention | | | ☐ Yes – surgical re-intervention | If applicable, both radiologically guided (eg drain) + surgical re-intervention may be selected |
| **If appendicectomy performed, macroscopic appearance** | ☐ Normal appendix | ☐ Simple appendicitis | | | ☐ Complex appendicitis | Macroscopic appearance – see operation note  Normal appendix: may be described ‘lily white’  Simple appendicitis: injected/ inflamed appendix that does not have ‘complex’ features  Complex appendicitis: perforated, purulent or necrotic appendix |
| **If appendicectomy performed, appendix histology** | ☐ Normal appendix | ☐ Simple appendicitis | | | ☐ Complex appendicitis |  |
|  | ☐ Adenocarcinoma | ☐ Carcinoid | | | ☐ Mucocele |  |
|  | ☐ Crohn’s Disease | ☐ Other | | | ☐ Pathology not done |  |
| **Complications of surgery if applicable** | ☐ Grade I  ☐ Grade I(d) | | ☐ Grade II  ☐ Grade II(d) | | ☐ Grade IIIa  ☐ Grade IIIa(d) | Please refer to appendix 1 to clarify definitions of each grade |
|  | ☐ Grade IIIb  ☐ Grade IIIb(d) | | ☐ Grade IVa  ☐ Grade IVa(d) | | ☐ Grade IVb  ☐ Grade IVb(d) |  |
|  | ☐ Grade V  ☐ Grade V(d) | |  | |  |  |

Appendix 1  Clavien Dindo Chart

| Grade | | Definition |
| --- | --- | --- |
| Grade I | | Any deviation from the normal postoperative course without the need for pharmacological treatment or surgical, endoscopic, and radiological interventions Allowed therapeutic regimens are: drugs as antiemetics, antipyretics, analgetics, diuretics, electrolytes, and physiotherapy. This grade also includes wound infections opened at the bedside. |
| Grade II | | Requiring pharmacological treatment with drugs other than such allowed for grade I complications |
| Grade III | | Requiring surgical, endoscopic or radiological intervention |
|  | Grade IIIa | Intervention not under general anaesthesia |
|  | Grade IIIb | Intervention under general anaesthesia |
| Grade IV | | Life-threatening complication (including CNS complications)* requiring IC/ICU management |
|  | Grade IVa | Single organ dysfunction (including dialysis) |
|  | Grade IVb | Multi organ dysfunction |
| Grade V | | Death of a patient |
| Suffix ‘d’ | | If the patient suffers from a complication at the time of discharge, the suffix “d” (for disability) is added to the respective grade of complication. This label indicates the need for a follow-up to fully evaluate the complication |

* Brain hemorrhage, ischemic stroke, subarrachnoidal bleeding, but excluding transient ischemic attacks, *CNS* central nervous system, *IC* intermediate care, *ICU* intensive care unit

**CENTRE SURVEY (To be completed once per site prior to data collection commencement)**

|  | Data Criteria | Options |
| --- | --- | --- |
| *Centre details* | | |
| 1 | Does you hospital care for? | ☐Adults only  ☐ Children only  ☐ Adults and children |
| 2 | Does your hospital have an on-site gynaecology service? | ☐ Yes  ☐ No |
| 3 | Does your centre have ‘review clinic’ slots for patients to return for further assessment/imaging the following day if a diagnosis is unclear? | ☐ Yes – with ultrasound and clinical review  ☐ Yes – clinical review only  ☐ No |
| 4 (a) | How many consultants will be “on call” during the first 2 week study period (13th-26th March)? | Number = |
| 4 (b) | How many consultants will be “on call” during the second 2 week study period (24th April- 7th May)? | Number = |
| 4 (c) | How many consultants will be “on call” during the third 2 week study period (5th-18th June)? | Number = |
| 4 (d) | How many consultant general surgeons work at your centre? | Number = |
| 4 (e) | Is there a dedicated surgical registrar based on SAU to review acute ED patientsreferrals? | ☐ Yes – 24/7  ☐ Yes – During the day  ☐ No – One registrar splits time between theatre and SAU |
| 5 | At weekends, Is ultrasound available? | ☐ Yes  ☐ No |
| 6 (a) | At weekends, is CT available? | ☐ Equivalent to weekday service  ☐ Reduced service but available for urgent surgical requests  ☐ Not available |
| 6 (b) | At night, is CT available? | ☐ Equivalent to weekday service  ☐ Reduced service but available for urgent surgical requests  ☐ Not available |
| *Does your centre have an agreed policy for* | | |
| 7 | When to use appendicitis risk stratification scores? | ☐ Yes – use of score recommended  ☐ Yes – use of score discouraged  ☐ No policy in place |
| 8 | Which patients should have a CT scan prior to appendicectomy? (e.g. diagnosis unclear, age >50) | ☐ Yes – please detail  ☐ No policy in place |
| 9 | Whether some patients with appendicitis may be managed non-operatively? | ☐ Yes –conservative management recommended for some patients; please detail  ☐ Yes – policy discourages conservative management  ☐ No policy in place |
| 10 | Whether laparoscopic or open appendicectomy should be routinely performed in adults? | ☐ Yes – open surgery recommended  ☐ Yes – laparoscopic surgery recommended  ☐ No policy in place |
| 11 | Whether laparoscopic or open appendicectomy should be routinely performed in children? | ☐ Yes – open surgery recommended  ☐ Yes – laparoscopic surgery recommended; if so, is it based on      ☐ Patient’s age          ☐ Patient’s weight  ☐ No policy in place  ☐ Not applicable – no paediatric surgery |
| 12 | Whether a macroscopically normal looking appendix should be removed or left in situ? | ☐ Yes – removal recommended  ☐ Yes – recommend it be left in situ  ☐ No – no policy in place |
